# Institutionalising community engagement for quality of care: moving beyond the rhetoric

**DOI:** 10.1136/bmj-2022-072638

**Published:** 2023-05-15

**Authors:** Brynne Gilmore, Paul Henry Dsane-Aidoo, Mikey Rosato, Nuhu Omeiza Yaqub, Roseline Doe, Sushil Baral

**Affiliations:** 1 School of Nursing, Midwifery and Health Systems, University College Dublin, Ireland; 2 World Health Organization, Ghana; 3 Women and Children First, UK; 4 Department of Maternal, Newborn, Child, Adolescent Health, and Ageing, World Health Organization, Geneva, Switzerland; 5 HERD International, Kathmandu, Nepal

## Abstract

Community engagement has the potential to improve quality of care but is poorly represented in policy and the literature; its institutionalisation in health systems must be supported, argue **Brynne Gilmore and colleagues**

**Key messages**

Comprehensive and appropriate community engagement is critical for quality improvement initiatives and improving health outcomes for communities
Too frequently community engagement is poorly defined and inadequately documented, and efforts to engage communities are not integrated into existing health systems
Experience from Ghana and Nepal shows the importance of generating and documenting evidence of impact as well as cost effectiveness to support institutionalisation of community engagement to improve health quality

Community engagement is widely recognised as a cornerstone of public health programming to achieve universal health coverage,^1^ ^2^ but what community engagement entails and whether it’s robustly implemented are not always clear. Here, community engagement refers to groups of people in communities collaborating with other stakeholders in the identification, planning, design, governance, and delivery of health services to tackle health related matters and promote wellbeing (box 1).^5^ ^6^

Box 1 **Relevant definitions for understanding the complexity of community engagement**

Community engagement terminology is used inconsistently. Similar terms are used to describe different approaches, and different terms are used to describe similar approaches. There are no consistently used definitions for key terms such as community, community engagement, community participation, or community mobilisation.^3^ For the purpose of this work, the following definitions and descriptions taken from relevant literature and author experience are used to help support an understanding of community engagement.

*Community*—The social boundaries that define the individuals and households whose health outcomes matter as a health system goal but also the social context for the relationships that underpin the success of many health system interventions.^4^

*Community engagement*—Communities collaborating with other stakeholders in the planning, design, governance, and delivery of health services to tackle health related matters and promote wellbeing.^5^ ^6^

*Approaches to community engagement*—In a 2020 report, the World Health Organization identified four categories of community engagement that can be used to define different levels of engagement: community orientated, community based, community managed, and community owned approaches.^2^

One way outreach, mobilisation without any community input, and communication activities that exclude reciprocal dialogue or feedback should not be considered community engagement because they lack meaningful engagement and exchange with communities. Although an external agent can initiate community engagement, it is not led, owned, or sustained by them. It is not a programme, reporting afterthought, or check box activity to falsely align work to “best practice.”

Community engagement initiatives are complex, existing on a continuum of less to more meaningful involvement of people who use the healthcare services in question and are not always clearly defined or described in the literature.^7^ Community engagement has been used to describe, for example, preliminary consultation with community members to identify or propose health interventions (less meaningful) and inclusion of communities in the identification, design, implementation, and evaluation of interventions (more meaningful). Community participation and community mobilisation are frequently used interchangeably with community engagement, with inconsistent definitions across these terms, which further complicates understanding.^7^

The benefits of involving communities in the design and planning,^8^ implementation, and delivery^9^ of health quality improvement interventions are clear. Evidence shows that community involvement can have a positive effect on health awareness, build community capacity to respond to health issues,^10^ and can improve the acceptability and ownership of communities and patients to health services.^11^ ^12^ Given that efforts to improve quality of care need to be responsive to the needs and preferences of communities,^5^ the process of engaging communities is essential for quality improvement.^13^ Unfortunately, despite its importance for key global health priorities such as universal health coverage, community engagement is poorly articulated in policy and academia and either fails to be meaningful for communities or is forgotten. Community engagement is also inconsistently or poorly integrated into quality of care policies and programmes. A 2019 policy survey, for example, found that only 60% of countries across all six geographical regions of the World Health Organization (range 47% to 72%) have a mechanism to solicit community feedback at health facilities on the quality and accessibility of services.^13^ Community engagement must be institutionalised, or integrated into government agendas and programmes as well as national health planning, to support quality improvement and to realise its benefits more broadly. We discuss examples from Ghana and Nepal to emphasise the importance of documenting the effects of community engagement to support its institutionalisation.

## Community engagement is essential for quality improvement

Although specific evidence is scarce and often imprecisely described, community engagement directly supports quality of care.^14^ Benefits include better health outcomes,^6^ ^15^ more equitable delivery of services,^16^ and enhanced community empowerment, ownership, and accountability.^17^ ^18^ In part, this is because community engagement ensures that interventions are specific to their context, as communities have input into identifying needs and delivery systems.^19^ A 2015 qualitative study in the Democratic Republic of the Congo, for example, found that using community scorecards as a form of community engagement improved key elements of quality of care, including access to services, relationships between communities and providers, and service provision.^20^ With respect to equitable service delivery, a 2023 mixed methods study examined knowledge, attitudes, and practices of Indigenous Mayan women in Guatemala (who are often marginalised) over three years of community engagement in a combined care group package, which sees communities identifying priorities and developing action plans along with the implementation of care groups and community birthing centres. It found significantly increased rates of participation in decision making and reported qualitative improvements in empowerment.^21^ Additional evidence indicates that community engagement can help make healthcare provision more people centred, by identifying and appropriately responding to community needs, for example.^5^ Community engagement can facilitate relationship building between communities and providers,^14^ which can encourage a positive experience for women in pregnancy and childbirth.^22^ A 2020 study from Ethiopia on women who regularly attended antenatal care but did not choose to give birth in a facility identified negative experiences with health providers as a driving factor.^23^ Community engagement can also support monitoring^14^ and can hold providers, policy makers, and community actors accountable for services and healthcare provision.^24^ ^25^ On the basis of this evidence, and recognising the ways in which community engagement is integral to quality improvement processes,^26^ tools^13^ and modules^27^ have been developed to support policy makers and implementers to integrate community engagement in quality of care improvement efforts.

Unicef has proposed quality standards and indicators for community engagement across four categories (core community engagement standards, standards supporting implementation, standards supporting coordination and integration, and standards supporting resource mobilisation).^28^ These include criteria around meaningful participation and its processes, policies, and approaches, and around how action plans and identification leaders and mobilisers should be developed with communities. The Network for Improving Quality of Care for Maternal, Newborn, and Child Health has recognised community engagement as core to quality improvement initiatives,^29^ and efforts to strengthen the institutionalisation of community engagement across its countries are ongoing.

Enthusiasm and advocacy for community engagement to drive quality improvement in maternal, newborn, and child health are abundant globally and nationally,^29^ but more robust documentation of community engagement processes, programmatic monitoring, and outcome reporting is needed,^30^ especially because concepts can be difficult to evaluate and compare.^31^

Community engagement can differ across and within programmes, and there is a risk that, without a clear understanding of what community engagement is and how to support its integration within policies and programming, poorly designed or superficial attempts at community engagement could result in less coordinated interaction between community actors and health service delivery counterparts. Institutionalisation of community engagement would mean having formal policy and implementation plans and structures supported by leadership and resources across various levels of service implementation—for example, establishing structures that see active community representation and representatives supported across the entire health system at the community, regional, and national levels. Financing and support for such work would be built into policies and frequently monitored. This, with robust reporting, evaluation, and accountability mechanisms in place, such as scorecards, published indices, or transparent and published expenditures, would ensure evidence based and sustained integration. Experience from Ghana and Nepal show how efforts to document the effects of community engagement on quality of care can embed it in national health service delivery planning.

## Community generated evidence in Ghana

A comprehensive national community engagement strategy can help institutionalise this critical component of quality improvement. Community engagement has been the bedrock of Ghana’s agenda towards universal health coverage, helping bridge gaps in healthcare access.^32^ The Community Based Health Planning Services (CHPS) for delivering primary care in Ghana was implemented because up to 70% of people lived more than 8 km away from a healthcare facility. Within CHPS, community health management committees (CHMCs) were formed to mobilise resources for facilities, support community health volunteers, strengthen the health system, and empower communities. Situated at the community level throughout the country, they are composed of people selected by community leaders and endorsed by community members and are associated with a specific facility. Because communities were engaged in establishing and scaling up the CHMCs, and because CHMCs are integrated in Ghana’s primary care through CHPS,^33^ the CHMCs have enhanced community involvement and ownership of primary healthcare.^34^ The National Quality Healthcare Strategy evolved as a result of learning from CHPS and broader community engagement and puts clients’ experiences at the centre of quality healthcare.^35^

During implementation of CHPS, a community scorecard was introduced for the community to provide formal feedback on quality of care in maternal, newborn, and child health to all healthcare levels. Every quarter, CHMC members engage with the wider community to discuss, score, plan, and monitor the health facility using predetermined quality of care indicators related to both provision and experience of care, including indicators such as waiting times, medical support availability, and provision of respectful and compassionate care. Scorecard results are entered into an online platform, which can be visualised for each facility, regions, and countrywide for decision making. After each scorecard assessment, health facility leaders and CHMCs develop a joint action plan to fill the gaps observed, and community representatives lead action plans for quality improvement in health facilities. Thus, in Ghana, community generated evidence showing improvements in quality of care has deepened community ownership of healthcare and continues to drive progress. The role of community engagement in the scorecards has been seen to elicit further support from policy makers and health officials in a positive feedback cycle that solidifies the role and importance of community engagement such that it becomes institutionalised.

Community voices therefore have a critical role in shaping healthcare policy.

## Community led solutions in Nepal

In the early 1960s, the recently democratised government in Nepal began focusing on selected health programmes to support community development. Over time, efforts to mobilise community health providers led to engagement initiatives, such as community level health promotion, and governance and social accountability activities including the female community health volunteer programme, mothers’ groups, and health facility operation management committees, which work collaboratively with communities to improve health. When found to be effective, these were scaled up, leading to increased access across the country to quality health services, especially for maternal, newborn, and child health and family planning programmes.^36^ Community engagement through health facility and operational management committees, for example, led to several positive changes in quality of care including improved skilled birth attendance and child growth monitoring.^37^ The gradually expanded and tested community engagement initiatives drove community led solutions to overcome barriers to quality of care in maternal, newborn, and child health.

Community engagement activities such as mothers’ groups and health facility and operational management committees expanded the reach and enhanced social accountability for marginalised and rural groups.^38^ They have improved health outcomes and have proved cost effective.^39^ ^40^ A 2020 systematic review of cost effectiveness of women’s group interventions found that, although staff costs represent about 77% of the total spending for these groups, scaling up to all rural regions in Nepal would cost 6.3% of total governmental health expenditure but would avert 15% of total neonatal deaths.^41^ The success of these community led solutions^42^ has solidified their role as core components of the community health system^43^; the cost effectiveness and promotion of equity facilitated government support of these community led solutions. Institutionalisation of community engagement is reflected in Nepal’s national health policies and strategies (1990, 2014, and 2019), which recognise community engagement as an established pillar of primary healthcare.

## Moving beyond rhetoric

Experiences with community engagement in Ghana and Nepal show the importance of regular evaluation and rigorous documentation to be fed into iterative policy making for solidifying the role of community engagement in health programming. Assessing both the cost effectiveness of community engagement activities and their effects on health equity can also support institutionalisation. It is important to recognise that institutionalising community engagement is not a quick process—it takes time and sustained investment. Other strategies to support the institutionalisation of community engagement can include recruiting community stakeholders, such as leaders or champions, from the onset of developing health initiatives^44^; using feedback mechanisms to inform and refine implementation^45^; and employing multiple coordinated initiatives targeted at a common goal but tackling different system levels or actors. Despite the importance of generating evidence to support and institutionalise community engagement, it continues to be poorly described and documented in the literature, and claims about the importance of community engagement are often not substantiated by evidence.^3^ ^7^ Understanding how, why, when, and for whom community engagement for quality of care can work is essential. Using common definitions and, when appropriate, standard indicators can support cross context learning and provide clarity to the field.

Ghana and Nepal are positive examples, but community engagement is often not a simple, linear, or straightforward process. In both countries, early official support of community engagement, an ability to make results of community engagement efforts visible to policy makers, and the governments’ readiness to adopt and adapt policies based on evidence of impact were critical. There are barriers to the institutionalisation of community engagement. Healthcare workers, including community health workers, who frequently operationalise community engagement activities are often over-worked, under-resourced, and unequally distributed across contexts.^46^ Institutionalisation efforts might therefore add additional work burden to community health workers, and failing to consider or manage their capacity could hinder efforts to institutionalise community engagement. Lastly, community engagement is neither easy nor cheap and requires a great deal of technical competency and resources. It is a dynamic and demanding process that needs appropriate support; but the pay-offs in health and society can be immense when done correctly. To provide people centred, high quality care that is responsive to the needs and contexts of communities, we must move beyond community engagement as rhetoric and re-focus on actions that support its institutionalisation.

## Notes

Contributors and sources: The authors are academics and practitioners working in community engagement or quality of care globally, most of whom have been involved with the Quality of Care Network. BG is an academic focusing on community engagement and quality of care; PDA is a health and quality of care specialist; MR is an expert in community based approaches; NOY is a health and quality of care specialist; RD is a health specialist; AP is a scientist focusing on community participation; SB is a health and development expert. BG, MR, NOY, and AP conceived the paper’s initial idea and key arguments. PDA, RD, and SB wrote the country case studies. All authors contributed to manuscript drafting and revisions. BG is the guarantor.

## Notes

Patient involvement: No patients were involved in this article.

This article is part of a collection proposed by the World Health Organization and World Bank and commissioned by The BMJ. The BMJ peer reviewed, edited, and made the decision to publish these articles. Article handling fees are funded by the Bill and Melinda Gates Foundation. Jennifer Rasanathan, Juan Franco, and Emma Veitch edited this collection for The BMJ. Regina Kamoga was the patient editor.

Funding: none.
